# 24th “Nantes Actualités en Transplantation” and 4th “LabEx Immunotherapy-Graft-Oncology” NAT and IGO Joint Meeting “New Horizons in Immunotherapy”

**DOI:** 10.3389/fimmu.2021.738312

**Published:** 2021-09-03

**Authors:** Noémie Joalland, Kathleen Ducoin, Gwenann Cadiou, Catherine Rabu, Carole Guillonneau

**Affiliations:** ^1^Nantes Université, CHU Nantes, INSERM, Centre de Recherche en Transplantation et Immunologie, UMR 1064, ITUN, Nantes, France; ^2^Université de Nantes, Inserm, CRCINA, Nantes, France

**Keywords:** immunotherapy, transplantation, autoimmunity, cancer, meeting report

## Abstract

The 24th edition of the annual NAT conference (Nantes Actualités Transplantation) and the 4th edition of the biennial LabEx IGO meeting (Immunotherapy Graft Oncology) were held jointly around a common theme: “New horizons in immunotherapy”, on May 31st and June 1st 2021 to highlight new findings in the fields of transplantation, autoimmunity and cancer.

## Overview of the Meeting

The 24th edition of the annual NAT conference (Nantes Actualités Transplantation) and the 4th edition of the biennial LabEx IGO meeting (Immunotherapy Graft Oncology) were held jointly and entirely online, on May 31st and June 1st 2021. For this edition, the two events decided to join their forces around a common theme: “New horizons in immunotherapy”, in order to offer an overview of recent advances in immunology, transplantation, autoimmunity and cancer. This NAT and IGO joint meeting have thus revealed the most advanced concepts and work of researchers and clinicians in the field of immunotherapies.

The conference program has featured 4 sessions with the 2 first sessions devoted to bioreagents and advanced cell therapy to control immune responses in the context of immunotherapy in transplantation, autoimmunity and cancer. The 3rd session was devoted to understanding new horizons in immunotherapy and the 4th session focused on tissue microenvironment and its role in the development of innovative immunotherapies.

## Session 1 – Bioreagents

The first session was chaired by Dr Paolo Dellabona (San Raffaele Institute, Milan, Italia) and Dr Carole Guillonneau (Centre de Recherche en Transplantation et Immunologie (CRTI), Nantes, France) and presented an overview of different strategies to regulate the immune system in cancer and transplantation through the use of bioreagents.

****Dr Eliane Piaggio, research director of the translational team at Institut Curie in Paris, works since many years on **IL-2 based immunotherapies for disengaging Tregs**. IL-2 based immunotherapy is a well-known strategy, used for decades, especially for cancer treatment but with complex issues. In fact, this cytokine is hard to use due to its systemic toxicity at high doses (e.g. cytokine storm) and its lack of specificity between T cell subtypes (effector T cells *vs* regulatory T cells) at low doses. Dr Piaggo’s team goal was to selectively target the regulatory component of the immune response in the tumoral microenvironment by using IL-2/anti-IL-2 mAb complexes (IL-2Cx). Depending on the epitope recognized by the anti-IL-2 mAb, this immune complex can preferentially act on Tregs, to control autoimmunity, or effector T cells, to induce anti-tumor response (1). *In vivo* experiments in a B16-C57BL/6 model proved the efficacy of IL-2Cx as a sole agent by reinvigorating exhausted intratumoral CD8^+^ T cells and also improved CTLA-4 blockade immunotherapy through immunomodulation of intratumoral Tregs that rescue NK cell antitumor function (2). In collaboration with Novartis, they are transferring IL-2Cx in human and exploring additional variants.

Antibody based immunotherapies and **how harnessing innate immunity in cancer therapy** was highlighted by Prof Eric Vivier, professor at the Centre d’Immunologie de Marseille-Luminy (CIML) in France and Scientific Director of the company Innate Pharma. For many years, immunomodulatory approaches have focused on immune checkpoint inhibitors or activating bispecific antibodies. This has led to unprecedented successes. However, many patients are not eligible for such strategies and resistance frequently appears. Many strategies target T cells but innate immunity plays an important role in cancer immunity. More specifically, NK cells, capable of direct cytotoxicity and secretion of immunomodulatory cytokines represent an attractive target (3). Immune checkpoint inhibitors specific for NK cells were generated such as Monalizumab blocking the NKG2A/HLA-E pathway and potentiating NK cell activation (4). NK cells engager composed of trifunctional molecules were designed to allow simultaneously activation of NKp46, ADCC (antibody dependent cell cytotoxicity) through CD16 and tumor antigen specific recognition (for example through CD20) (5). Interestingly, NK cell engagers are more efficient than the combination of individual mAbs and bispecific mAbs. Several pharmaceutical companies develop such kind of molecules and clinical trial are starting, predicting a new wave of efficient anti-tumor immunomodulatory molecule.

Among anti-tumor strategies another big way is to target directly tumor cells to kill them. Dr Jean-François Fonteneau****, researcher at the Centre de Recherche en Cancérologie et Immunologie Nantes Angers (CRCINA, France) presented an antitumor virotherapy strategy based on **oncolytic activity of attenuated Measles virus and defect in the type I interferon response in cancer**. Oncolytic viruses represent interesting tools because they infect dividing cells, such as tumor cells, and their replication leads to immune cell death of infected cells which stimulates anti-tumor responses. From an immunotherapeutic perspective, non-pathogenic attenuated Measles Virus (MV) can be used thanks to their spontaneous oncolytic activity and use of CD46 as entry receptor which is often overexpressed by tumor cells, especially in malignant pleural mesothelioma (MPM). After establishing a primary cell line bank of MPM, they showed that tumor cells were not all sensible to oncolytic activity of MV. Susceptibility to infection is due to a defect of intracellular type I interferon pathway in infected cells, mainly involved in antiviral response (6). They demonstrated that this sensitivity is due to a homologous deletion of IFN I encoding genes linked to their localization on the same chromosomic region of CDKN2A tumor suppressor gene, frequently deleted in cancer (7). They also set up a physiological multi-cellular tumor spheroid model to study the influence of non-malignant cells on MV oncolytic activity (fibroblast and endothelial cells) (8).

To finish this session, Dr Jordi Orchando****, Assistant Professor of Oncological Sciences at the Icahn School of Medicine at Mount Sinai in New York, described the **impact of trained immune cells in tolerance and organ transplantation**. Immunological memory is an old and well-known characteristic attributed to the adaptive immune system, but recent studies also demonstrated the existence of trained immunity which is a form of innate immune memory. Proofs of trained immunity were revealed by non-specific protection to Bacille Calmette-Guerin during vaccination (9). This memory originated in epigenetic changes of inflammatory macrophages during their restimulation and more specifically an enhancement of H3K4 methylation which allow higher gene expression (10). So, trained immune cells represent interesting target for immunotherapy. Then, a strategy based on mTOR inhibitor encapsuled in HDL nanoparticle (mTORi-HDL) was presented with demonstration of efficacy in an experimental heart transplant model of mice. mTORi-HDL targets trained macrophages infiltrating the graft to prevent activation and promote organ transplant acceptance with no sign of toxicity and vasculopathy (11). Finally, the interesting and not common question of cancer in organ transplant patients was also addressed.

## Session 2 – Advanced Cell Therapy

Session II was chaired by Dr Dimitrios Wagner (Charité Universitätsmedizin, Berlin, Germany) and Dr Nathalie Labarrière (CRCINA, Nantes, France) and focused on advanced cell therapies in auto-immune diseases and cancer.

Dr Silvia Gregori****, research director at the San Raffaele Telethon Institute for Gene Therapy in Milan, Italy, focused her talk on** dendritic cells (DCs) and their impact in autoimmune diseases and transplantation**. They identified a subset of tolerogenic DCs producing IL-10 and named DC-10. Since they highlighted a diminution of DC-10 in patients’ blood exhibiting autoimmunity (in Type 1 diabetes and Multiple sclerosis models) or severe inflammation (in Celiac disease model), DC-10 could be defined as biomarker of tolerance and used in clinical applications (12). To support this idea, they induced, with lentiviral constructs, human monocyte-derived DCs producing IL-10 *in vitro* and capable of maintaining their phenotype and functionality in humanized mice (13). They optimized their constructs to generate DC-10 overexpressing MHC class II antigens (Ag) which might induce Ag-specific Tr1 cells, modulate Ag-specific CD4^+^ T cells and allo-specific CD8^+^ T cells and promote T cells with an exhausted phenotype. To conclude, DC-10 could have the capacity to reverse the trend in autoimmunity (or inflammation) and DC-10-based therapy would restore tolerance in these diseases.

Prof Stanley Riddell**** (MD, PhD, Fred Hutchinson Cancer Research Center, Seattle, USA) was one of the first to perform proof of concept studies showing that antigen specific T cells could be used to boost T cell immunity against viruses and to tackle cancer. He is also a pioneer in using engineered and modified CAR or TCR transduced T cells in human. He gave a much-appreciated talk about **overcoming barriers to efficacy for engineered T cells in solid tumors** during which he presented results describing a CD4 T cell population directed against a neoantigen derived from the oncogenic mutated BRAF^V600E^ gene expressed in melanoma cell. This population was identified within the Tumor Infiltrating Lymphocytes (TIL) population adoptively transferred to a stage IV acral melanoma patient (14). Prof Riddell also presented results on the identification of CD4 T cell responses to mutation-encoded neoepitopes in lung cancer (15). Then, using an approach of single cell RNAseq coupled to specific TCR sequencing, he was able to deeply characterize the transcriptional signature of T cells that are truly tumor reactive within the TIL population. In particular, he presented unpublished results indicating that the reactive cells shared many features with T follicular cells. This precise characterization of reactive T cells will help to define cellular markers to aim for in therapeutic settings.

The next speaker was Dr Alexander Marson**** (MD, PhD) who is an associate professor at UCSF (USA). He presented a strategy that he developed to efficiently ******reprogramming the genome of human immune cell populations using the CRISPR/Cas9 technology**. He used this global strategy with a guide library in order to identify relevant targets for modulating T cell responses. In particular, Dr Marson found that RASA2, which is a GTPase-activating Protein (GAP) functioning as a negative regulator for Ras, was a promising target for novel immunotherapeutic strategies (enhanced division capacities, enhanced tumor cell killing when invalidated) (16). Knocking out RASA2 gene (CRISPR/Cas9) had broad signaling effect and led to enhanced proliferation of T cell in response to stimulation, in particular in suppressive conditions. This approach could be used in various therapeutic settings such as CD19 CAR T cells or to avoid loss of activity after chronic antigen exposure.

The keynote lecture was given by Prof. John Wherry**** chair of the department of Systems Pharmacology and Translational Therapeutics at University of Pennsylvania (Philadelphia, USA). One of his area of interest is **molecular understanding of human T cell exhaustion and their role in disease**. He tries to understand which T cell population best correlates with clinical outcome in anti-tumor immunotherapy and which is critical to predict and optimize therapeutic responses. He presented results defining a precise human T cell molecular reference atlas. By combining two high throughput approaches (ATAC seq and RNA seq) he provided very detailed analysis of specific gene expression. The work was primarily performed by Josephina Giles and is about to be published.

## Session 3 – New Horizons

Session III was chaired by Prof Megan Sykes (Columbia Center for Translational Immunology, New York, USA) and Dr Catherine Rabu (CRCINA, Nantes, France) and devoted to new horizons in immunotherapy.

The session started with Dr Nicolas Dumond****, postdoctoral researcher at the University of Zurich, a specialist of **tissue ecosystem profiling by Imaging Mass Spectrometry** (IMC). He has a long-standing interest in the study of type 1 diabetes with the objective of understanding the progression of this auto-immune disease by reconstructing evolution of β cells and islet-infiltrating immune cells. To deeply profile this tissue ecosystems, he used IMC, a high-resolution technology coupling a laser ablation system with a time-of-flight mass spectrometry, allowing simultaneous analysis of spatial mapping and cell phenotyping through the detection of over 40 markers. Thanks to this technique, he realized an immune cell profiling on pancreas section from donors at different stages of disease progression: non-diabetic, pre-diabetic with autoantibodies, recent onset and long duration. This longitudinal study revealed that T cells infiltration was dependent and correlated to the islet profile, originated by alteration of β cell phenotype at pre-diabetic stage (17). He also evaluated the β cell response to treatment, its influence on neighbor cells, and developed a spheroid model reproducing the interplay of environment and cell state (18). In conclusion, this multi-level analysis helps understanding cell interactions inside their ecosystem and leads to a reconstruction of probable disease evolution, a strategy applicable to other diseases such as tumoral microenvironment.

Dr Karin Tarte****, director of a research unit dedicated to basic and translational research in the field of lymphoma at the University of Rennes, France, continued the session with **mesenchymal stromal cell (MSC) heterogeneity and its implication for their clinical application**. MSC have clinical potential based on their immunosuppressive and anti-inflammatory properties but their large heterogeneity could impact clinical responses. She draw our attention to the different levels of heterogeneity, starting with the variability due to the donor which requires strong phenotypic characterization (19) and the need of better understanding of human immune stroma precursors (20). Production process, which includes culture expansion and cryopreservation, also impacted cell functions, supporting the need for important laboratory standardization (21, 22). The last, but not least, aspect she described was the recipient-related heterogeneity and the next frontier before starting a clinical trial. Monitoring process for patient evaluation will rely on the identification of activity biomarkers and the design of precise potency assays to predict *in vivo* efficacy in patients (23).

The next speaker, Dr Laura Jardine comes from Newcastle University, UK and is Academic Clinical Lecturer in Hematology. She mainly works on bone marrow transplantation and presented a **single-cell genomic project for reconstructing the developing blood and immune system**. She took part in setting up a single cell atlas of human fetal bone marrow, available online: the Human Cell Atlas Developmental (https://www.humancellatlas.org/dca/). In a first step, she realized a multiomic analysis of fetal bone marrow, combining RNAseq and CITEseq, to identify and annotate each cell types (24). This analysis revealed a rapid diversification of myeloid cells associated with an expansion of B lymphocytes and NK cells. Her final goal was to use this cell atlas to compare single cell leukemia to normal cell transcriptomes to define the developmental state of infant B leukemias and to identify cancer-specific pathways to target. Her team also contributes to an international consortium to construct 3D reference anatomical structures, cell types and biomarkers tables based on the Human Cell Atlas (25).

Dr Christopher Klebanoff, the last speaker of this session, is both medical oncologist and translational researcher at Memorial Sloan Kettering Cancer Centre and the Parker Institute for Cancer Immunotherapy, NY, USA. He presented us how to **target common epithelial malignancies with TCR engineered lymphocytes specific for “public neoantigens”**. One challenge was to find an antigen as tumor target with minimum off target toxicities, homogenous expression and reduced susceptibility to acquired resistance. To this aim, his lab worked for years to identify public neoantigens, shared by a majority of patients and strongly conserved. Using mass spectrometry and single cell analysis, they identified a mutant of PI3K, a common human driver oncogene, expressed on patients with metastatic solid cancer (26). Moreover, a selective loss of heterozygote expression of HLA-A3 in tumor tissue was observed as an immune resistance mechanism (no recognition by NK cells) but lead to constitutive expression of this neoantigens by tumor cells. Then, they retrieved and cloned a library of TCR that individually conferred to transfected T cells specificity to this immunogenic shared public neoantigen with lack of cross reactivity (26). Interestingly, some of these TCRs can be functional in both CD4 and CD8 T cells. In conclusion, they have developed neoantigens as an innovative translational tool that can be applied to a number of different purposes.

## Session 4 – Tissue Microenvironment

The last session was chaired by Prof Barbara Seliger (Institute for Medical Immunology Martin Luther, University Halle-Wittenberg, Germany) and Dr Jérôme Martin (CRTI, Nantes, France) and focused on tissue microenvironment in cancer or in inflammatory situations.

Prof. Renato Monteiro, researcher and team leader at the Centre de recherche sur l’inflammation (INSERM U1149 & CNRS ERL8252) in Paris, France, began with Fc receptors (FcRs) and their **immunoregulation capacity of inflammation**. FcRs are mainly activating receptors, thanks to immunoreceptor tyrosine-based activation motif (ITAM), and participate in many functions in both innate and adaptative immunity. Nevertheless, FcRs bearing inhibitor ITAMs (ITAMi) have been highlighted and might play a role in homeostasis. As opposed to classical ITAM signaling, ITAMi signaling involves the recruitment of diverse inhibitory effectors, like SHP-1, by inducing an intracellular co-aggregation forming clusters termed “inhibisomes” (27). This recruitment occurred only when there are monovalent interactions between the ligand and the receptor, but not with multimeric interactions. Further study of the ITAMi pathway will provide a better understanding of inflammation regulation and its potential targeting.

**Myeloid cells** represent a diverse group of cells with multiple roles that can act in **promoting tumor growth**. Dr Antonio Sica (Humanitas Clinical and Research Center, Milan, Italy) gave an overview of his recent work in this field. He defined the concept of emergency myelopoiesis and showed in mice models that the RORC1 (retinoic-acid-related orphan receptor)-expressing myeloid cells are enriched in advanced tumors. MDSC (myeloid derived suppressor cells) and TAM (Tumor associated macrophages) expressing RORC1 promote cancer development (28). He also showed that in tumor, an enhanced production of Nicotinamide Phosphoribosyltransferase (NAMPT) leads to immature MDSC mobilization, representing a potential therapeutic target for enhanced anti-tumor activity (29). Finally, he described a subset of TAM (F4/80^high^) which overexpress HO-1 (heme-oxygenase-1) that plays a critical role in promoting tumor metastasis and immunosuppression. Interestingly, in metastatic melanoma patients, HO-1 expression on various monocyte populations discriminates survival as a good prognostic marker in case of low expression (30). To conclude, HO-1^+^ myeloid cells represent a new prognostic indicator and a novel antimetastatic target.

The next speaker, Prof Ofer Mandelboim, professor of Molecular Immunology at The Hebrew University, Jerusalem, Israel, presented his work on **TIGIT and its cellular and bacterial ligands, new checkpoints for cancer immunotherapy**. TIGIT is an inhibitory receptor present on Natural Killer (NK) and T cells and it interacts with *Candida* fungal pathogens. Since the 1980s, it has been shown that NK cells have abilities to recognize and kill transformed and virus-infected cells and also extracellular pathogens, such as *Candida*, responsible of various diseases (from Candida vulvovaginitis to invasive candidiasis). On that context, he presented data describing the first fungal ligands that impair NK cell functions through TIGIT and establishing the role of NK cells in the control of *C.albicans* infection.

Even if immunotherapies revolutionized antitumoral treatments, many patients are still not responding to these approaches. Prof. Barbara Seliger, director of the Institute of Medical Immunology, Martin Luther University at Hall-Wittenberg in Germany, described **immune escape mechanisms of tumors and their impact for immunotherapies**. She mostly described defects in the HLA class I antigen processing and presentation machinery (APM), occurring by mutations or (post) transcriptional regulations observed in many cancers at variable frequencies (from 30 to 80%). An altered APM in tumors was associated with a worst prognosis, like in oral squamous cell carcinoma, mainly due to a reduced intratumoral density of CD8 T cells (31). Using *in silico* prediction, they identified two microRNAs (miR), miR-26b-5p and miR-21-3p, involved in downregulating TAP1 protein expression thereby inducing a reduced HLA class I expression in melanoma cells (32). Moreover, hsa-miR-21-3p was qualified as an oncogenic miRNA since it was overexpressed in many cancers and able to diminish HLA class I expression. These observations highlighted the possibility to target miRNA in HLA class I^low^ tumors, to counter this immune escape mechanism.

The last talk of the session was given by Dr Shannon Turley, Staff Scientist in the Department of Cancer Immunology at Genentech in San Francisco. Her area of interest is about the **evolution of the stroma-immune axis in cancer immunology and immunotherapy**. She noted the beneficial therapeutic effects of blocking antibodies targeting the PD-1/PD-L1 pathway in bladder cancer, while emphasizing that anti-PD-L1 treatments (atezolizumab) were only active in subsets of patients (33). Using a transcriptomic analysis, she highlighted that TGF-ß pathway related genes were enriched in advanced bladder tumors from patients non responding to atezolizumab. In a combotherapy using anti-PD-L1 and anti-TGFß on a mammary carcinoma murine model, she showed an expansion of CD8 T memory progenitors and reduction of CD8 precursor exhausted (Tpex) T-cells (34). In a second step, performing scRNAseq of human pancreatic ductal adenocarcinoma (PDAC) and normal adjacent tissue, she described that 2 major cancer associated fibroblasts (CAF) clusters, including a TGFß-activated subtype in which Lrrc15, a mesenchymal protein induced by TGF-ß (35), was highly enriched. Furthermore, it appeared that a LRRC15/TGF-ß gene signature was associated with reduced survival and lack of response to atezolizumab (36). In conclusion, immunotherapy blocking both PD-1/PD-L1 and TGF-ß pathways would allow to stimulate tumor-specific immune responses together with targeting immunosuppressive tumor stroma.

## Conclusion

This 24th edition of the NAT conference and 4th edition of the LabEx IGO meeting took place online with more than 200 attendees and allowed interesting exchanges between international specialists in their field around the common theme of new horizons in immunotherapy. The exhaustive program included presentations of latest technical advances used to better understand physiopathology of transplantation, cancer, autoimmune diseases and highlighted the limitations still faced in these diseases. The congress also gave an overview of new active/passive immunotherapies developed based on molecular/cellular compounds to overcome failure of current treatments and to successfully treat patients in the future thanks to shelf cell therapy, genetic engineering, bioreagents and a better understanding of tissue ecosystem interactions in diseases.

## Author Contributions

All authors listed have made a substantial, direct, and intellectual contribution to the work and approved it for publication.

## Conflict of Interest

The authors declare that the research was conducted in the absence of any commercial or financial relationships that could be construed as a potential conflict of interest.

## Publisher’s Note

All claims expressed in this article are solely those of the authors and do not necessarily represent those of their affiliated organizations, or those of the publisher, the editors and the reviewers. Any product that may be evaluated in this article, or claim that may be made by its manufacturer, is not guaranteed or endorsed by the publisher.
